# Supplementary material for: Quality of life among pregnant women with urinary incontinence: A cross-sectional study in a Malaysian primary care clinic
Source: PLoS One. 2021 Apr 28;16(4):e0250714. doi: 10.1371/journal.pone.0250714 (PMC8081257; doi:10.1371/journal.pone.0250714)
Supplement: S1 File — (PDF) [file pone.0250714.s001.pdf]

STUDIES ABOUT KNOWLEDGE, FACTS AND PRACTICES OF PELVIC ERXERCISE (KEGEL) AMONG PREGNANT WOMEN, URINARY INCONTINENCE AND YOUR QUALITY OF LIFE.

Dear mothers, kindly complete this questionnaire. The purpose of this study is to determine the urinary incontinence and level of knowledge, attitude and practice among pregnan women on pelvic exercise (Kegel exercises), urinary incontinence and your quality of life.

Kindly answer all questions honestly and advise not to think long to answer questions. Please answer the questions according to the instructions in each section.

All information in this form is strictly confidential. Your kind co-operation is greatly appreciated.

A .Personal Data.

1. Respondent number: ____
2. Age:____(year)
3. Phone: (Home) ____________________________________
   (Mobile) ____________________________________
4. Race: Malay ☐ Indian ☐ Chinese ☐ Others ☐
5. Occupation: ____
6. Household income (per month):____________________
7. Educational level:
   i. Never been to school ☐
   ii. Primary School ☐
   iii. Secondary Islamic School ☐
   iv. Secondary Government School ☐
   v. Diploma/University ☐

B. Obstetric and Gynecology History

8. Current pregnancy is number _____________
9. How many live birth _______________________

12. Method delivery
   i. Normal delivery _______ times
   ii Vaccum _______ times
   iii. Forcep _______ times
   iv. Cesarean section _______ times

13. Body Mass Index (BMI):
14. Current weeks of pregnancy
